# The TRIB3 R84 variant is associated with increased left ventricular mass in a sample of 2426 White individuals

**DOI:** 10.1186/s12933-021-01308-4

**Published:** 2021-05-29

**Authors:** Gaia Chiara Mannino, Carolina Averta, Teresa Vanessa Fiorentino, Elena Succurro, Rosangela Spiga, Elettra Mancuso, Sofia Miceli, Maria Perticone, Angela Sciacqua, Francesco Andreozzi, Giorgio Sesti

**Affiliations:** 1 Department of Medical and Surgical Sciences, University “Magna Graecia” of Catanzaro, Viale Europa, 88100 Catanzaro, Italy; 2 Department of Clinical and Molecular Medicine, Sapienza University of Rome, Viale Regina Elena, 324, 00161 Rome, Italy

**Keywords:** Cardiovascular disease, Genetics, Insulin signaling, Left ventricular mass index, TRIB3 Q84R variant, rs2295490

## Abstract

**Background:**

Prior studies in animal models showed that increased cardiac expression of TRIB3 has a pathogenic role in inducing left ventricular mass (LVM). Whether alterations in TRIB3 expression or function have a pathogenic role in inducing LVM increase also in humans is still unsettled. In order to address this issue, we took advantage of a nonsynonymous *TRIB3* Q84R polymorphism (rs2295490), a gain-of-function amino acid substitution impairing insulin signalling, and action in primary human endothelial cells which has been associated with insulin resistance, and early vascular atherosclerosis.

**Methods:**

SNP rs2295490 was genotyped in 2426 White adults in whom LVM index (LVMI) was assessed by validated echocardiography-derived measures.

**Results:**

After adjusting for age and sex, LVMI progressively and significantly increased from 108 to 113, to 125 g/m^2^ in Q84Q, Q84R, and R84R individuals, respectively (Q84R vs. Q84Q, P = 0.03; R84R vs. Q84Q, P < 0.0001). The association between LVMI and the Q84R and R84R genotype remained significant after adjusting for blood pressure, smoking habit, fasting glucose levels, glucose tolerance status, anti-hypertensive treatments, and lipid-lowering therapy (Q84R vs. Q84Q, P = 0.01; R84R vs. Q84Q, P < 0.0001).

**Conclusions:**

We found that the gain-of-function *TRIB3* Q84R variant is significantly associated with left ventricular mass in a large sample of White nondiabetic individual of European ancestry.

## Background

Increased left ventricular mass (LVM) as determined by echocardiography is an organ damage that has been associated with cardiovascular morbidity and mortality [1, 2]. The pathophysiological mechanisms underlying LVM increase are complex and multifactorial involving adaptative cardiac remodelling to blood pressure overload as well as genetic [3, 4], anthropometric [5, 6], hormonal [7–9], and metabolic factors [10–13]. Amongst the latter, most [5–7, 14–20], but not all studies [21, 22], have shown that insulin resistance and compensatory hyperinsulinemia may have a role in the pathogenesis of increased LVM. The molecular mechanism linking insulin resistance/hyperinsulinemia to altered myocardial structure remains incompletely understood. Preclinical studies carried out in cellular and animal models have shown that cardiac insulin resistance is associated with an impairment in the insulin signalling cascade involving the activation of phosphatidylinositol3-kinase (PI3K) and its downstream substrate, protein kinase B (Akt) upon binding of insulin to its receptor, and phosphorylation on tyrosine residues of IRS-1/2 proteins [23]. There is evidence that altered expression of negative regulators of Akt expression and activity such as phosphatase and tensin homologue (PTEN) [24], protein tyrosine phosphatase 1B (PTP1B) [25], the PH domain leucine-rich repeat protein phosphatase (PHLPP) [26], and tribbles homologue 3 (TRIB3) [27] may have a causative role in impairing the insulin signalling pathway. Amongst these Akt inhibitors, TRIB3 is a plausible candidate for linking molecular insulin resistance to LVM increase for the following reasons: (a) a rat model of type 2 diabetes induced by a combination of high-fat diet and low-dose streptozotocin exhibits insulin resistance, cardiac hypertrophy, and increased cardiac expression of TRIB3, as compared with control rats [28]; (b) silencing of TRIB3 in these diabetic rats restores Akt activity, reduces the phosphorylation of extracellular signal–regulated kinase 1/2, improves insulin resistance, and attenuates myocardial hypertrophy [28]; (c) dietary supplementation with Zn in *db/db* type 2 diabetic mice increases expression of the antioxidant metallothionein (MT), which results in reduction in TRIB3 expression accompanied by increased Akt2 activity, and protection from diabetes-induced cardiac structural and functional changes including LVM increase [29]. Whether alterations in TRIB3 expression or function have a pathogenic role in inducing LVM increase also in humans, is still unsettled. In order to address this issue, we took advantage of a nonsynonymous *TRIB3* Q84R polymorphism (rs2295490), a gain-of-function amino acid substitution impairing insulin signalling, and action in primary human umbilical vein endothelial cells (HUVECs) naturally carrying the *TRIB3* Q84 or R84 variant [30, 31], which has been associated with insulin resistance, and early vascular atherosclerosis [31, 32]. Unfortunately, neither the TRIB3 Q84R polymorphism, nor any other SNP in good linkage disequilibrium with it, has been included in the arrays used in the publicly available genome-wide association studies (GWAS) for left ventricular mass, thus precluding the possibility of performing in silico analyses. However, in a recent meta-analysis of three GWAS including hypertrophic cardiomyopathy cases of European ancestry from the Netherlands, the United Kingdom and Canada, SNP rs6115789, a good proxy of the *TRIB3* Q84R polymorphism, was found to be associated with hypertrophic cardiomyopathy in Dutch individuals (*P* = 6 × 10^–4^), indicating the need for further studies to address the role of the *TRIB3* Q84R polymorphism in LVM [33].The current study was therefore undertaken to assess the impact of Q84R TRIB3 variant on LVM in a large group of individuals participating in the CATAMERI study, an ongoing observational study recruiting adult subjects with one or more cardiometabolic risk factors who underwent a complete clinical characterization including standard Doppler echocardiography [34].

## Methods

### Study subjects

The study group consisted of 2426 White individuals participating in the CATAMERI study, an observational study of adults with one or more cardiometabolic risk factors recruited at the university hospital of the University “Magna Graecia” of Catanzaro as previously described [34, 35]. The inclusion criteria were: age > 18 years and presence of one or more cardio-metabolic risk factors including dysglycemia, hypertension, dyslipidemia, overweight/obesity, and family history of diabetes. Subjects were excluded if they had diabetes mellitus, defined as fasting plasma glucose > 126 mg/dl or 2-h post-load plasma glucose > 200 mg/dl, current treatment with glucose-lowering agents or self-reported history of a previous diagnosis, end-stage renal disease (ESRD), chronic gastrointestinal diseases, liver cirrhosis, acute or chronic pancreatitis, acute or chronic infections, history of malignant or autoimmune diseases, history of alcohol or drug abuse, positivity for antibodies to hepatitis C virus (HCV) or hepatitis B surface antigen (HBsAg), and treatment with drugs known to influence glucose tolerance, such as steroids and estroprogestins employed for hormonal contraception or replacement treatment. All participants underwent anthropometrical evaluation including assessment of body mass index (BMI), waist circumference, and blood pressure. Systolic blood pressure and diastolic blood pressure were recorded at the first appearance (phase I) and the disappearance (phase V) of Korotkoff sounds. Blood pressure values were the average of the last two of three consecutive measurements obtained at intervals of 3 min. After an overnight fast, a 75-g OGTT was performed in subjects with fasting plasma glucose < 126 mg/dL (< 7 mmol/l), and no history of diabetes, and a venous blood sample was drawn for laboratory determinations. According to the American Diabetes Association (ADA) criteria [36], subjects were classified as having normal glucose tolerance (NGT) when fasting plasma glucose was < 100 mg/dl (5.5 mmol/l), 2-h postload glucose < 140 mg/dl (< 7.77 mmol/l), and HbA1c < 5.7%, and prediabetes when fasting plasma glucose was 100–125 mg/dl (5.5–6.9 mmol/l), or 2-h postload glucose was 140–199 mg/dl (7.77–11.0 mmol/l) or HbA1c 5.7–6.4%.

Pulse pressure was calculated as the difference between systolic blood pressure and diastolic blood pressure. The rate pressure product was calculated as heart rate × systolic blood pressure. The HOMA-IR index was calculated as fasting insulin × fasting glucose/22.5. Estimated glomerular filtration rate (eGFR) was assessed by using the MDRD equation: eGFR = 175 × (Scr)-1.154 × (Age)-0.203 × (0.742 if female) where Scr is serum creatinine [37].

The study was approved by the Institutional Ethics Committee of the University “Magna Graecia” of Catanzaro (approval code: 2012.63). Written informed consent was obtained from each subject in accordance with the principles of the Declaration of Helsinki.

### Echocardiographic assessments

Tracings were taken with individuals in a partial left decubitus position using a VIVID-7 Pro ultrasound machine (GE Technologies, Milwaukee, WI, USA) with an annular phased array 2.5-MHz transducer. Tracings were recorded under two-dimensional guidance, and M-mode measurements were taken at the tip of the mitral valve or just below. Measurements of interventricular septum thickness (IVS), posterior wall thickness (PWT) were made at end-diastole. LV end-diastolic (LVEDV) and end-systolic volume (LVESV) were measured according to Simpson method and indexed for body surface area (BSA) [38]. LV mass (LVM) was calculated using the Devereux formula [39] and normalized by BSA (LVMI) [38, 40].

### Analytical determinations

Glucose, triglycerides, total and HDL cholesterol concentrations were determined by enzymatic methods (Roche, Basel, Switzerland). Plasma insulin concentration was determined with a chemiluminescence-based assay (Immulite, Siemens, Italy).

### Genotyping of *TRIB3* gene polymorphism

Blood samples were collected from all patients. DNA was extracted from whole blood using commercial DNA isolation kits (Promega, Madison, WI and Roche, Mannheim, Germany). rs2295490/Q84R *TRIB3* genotype calls were determined with TaqMan allelic discrimination assay (Assay ID# C__16190162_10; Applied Biosystems, Foster City, CA), the DNA was amplified and fluorescence was detected on an iCycler Thermal Cycler with CFX384 Touch Real-Time PCR Detection System (Bio-Rad Laboratories, Inc., Hercules, CA). Good genotyping quality was ensured by including 0.05 ng of custom oligo strings (GeneArt® Strings™ DNA Fragments, Invitrogen, Thermo Fisher Scientific) with a sequence designed to span symmetrically ~ 200 bp around the context sequence of the genotyping assay, differing only for the rs2295490 allele A or G. The oligo strings were combined and loaded as three individual samples representing one heterozygous A/G and two sets of homozygous A/A and G/G controls, in each 384-well plate. Genotyping concordance of the oligo strings was 100%.

### Statistical analysis

Log transformation was used when analyzing triglycerides levels because their distribution did not respect the assumption of normality. Each SNP was coded as 0, 1, or 2 depending on the number of risk alleles in the patient. Smoking habit was coded as 0 = never smoked and 1 = current or former smoker. Continuous variables are expressed as means ± SD. Categorical variables were compared by χ^2^ test. Comparisons between the three genotypes were performed using a general linear model with post hoc Fisher's least significant difference correction for pairwise comparisons. The Hardy–Weinberg equilibrium between the genotypes was evaluated by *χ*^2^ test. All tests were two-sided. Power calculations were performed with Quanto version 1.2.4. The study had 86% power (for α = 0.05) to detect a 4 g/m^2.7^ change in LVMI per allele G according to an additive model. Associations between the TRIB3 Q84R polymorphism and LVMI are presented as effect sizes (β and SE) per copy of minor allele estimated by linear regression models adjusted for a number of confounders. We report nominal *P* value < 0.05 without adjustment for multiple testing given the high prior probabilities for association of the rs2295490 polymorphism with LVM. All calculations were done with SPSS software (program Version 22.0) for Windows.

## Results

The clinical characteristics of the study group are summarized in Table 1. The study group consisted of 2426 adult individuals (1182 men and 1244 women) with mean age of 51 ± 23 years and mean BMI = 29.6 ± 6.0 kg/m^2^.

**Table 1:** Anthropometric and metabolic characteristics of the study subjects stratified according to TRB3 genotype

Variables	Whole study group N = 2426	Q84Q N = 1792	Q84R N = 573	R84R N = 61	*P*
Gender (M/F)	1182/1244	864/928	289/284	29/32	0.640
Age (years)	51 ± 14	51 ± 14	52 ± 15	52 ± 14	0.270*
BMI (Kg/m^2^)	29.73 ± 6.0	29.64 ± 6.04	29.52 ± 5.93	29.73 ± 7.37	0.909
Waist circumference (cm)	100.5 ± 14.2	100.6 ± 14.3	100.4 ± 14.1	98.5 ± 12.1	0.429
Smoking status (never/current/ex) (%)	1792/573/61	1021/367/404	407/115/144	40/10/11	0.451
SBP (mmHg)	133 ± 18	133 ± 19	133 ± 18	137 ± 20	0.204
DBP (mmHg)	81 ± 11	81 ± 11	81 ± 11	85 ± 11	0.058
Pulse pressure (mmHg)	51 ± 14	51 ± 14	52 ± 14	51 ± 14	0.891
Heart rate (beats min^−1^)	71 ± 10	70 ± 10	71 ± 11	70 ± 10	0.179
Rate pressure product (mmHg × beats min^−1^)	9443 ± 2014	9403 ± 2019	9532 ± 1978	9783 ± 2164	0.271
Tot-Col (mg/dl)	201 ± 40	202 ± 41	197 ± 39	204 ± 35	0.106
HDL-Col (mg/dl)	52 ± 14	52 ± 14	51 ± 14	53 ± 15	0.709
Triglycerides (mg/dl)	128 ± 78	129 ± 78	127 ± 79	123 ± 57	0.408
Fasting glucose (mg/dl)	93 ± 10	93 ± 10	93 ± 11	92 ± 8	0.720
Fasting insulin (µU/ml)	10.2 ± 4.1	9.9 ± 3.9	10.2 ± 4.1	11.8 ± 4.4	0.02
HOMA-IR	2.33 ± 0.96	2.28 ± 0.94	2.34 ± 0.97	2.68 ± 1.01	0.03
eGFR (ml/min/1.73 m^2^)	97 ± 22	97 ± 22	96 ± 22	95 ± 27	0.128
NGT/Prediabetes	1508/918	1110/682	353/220	45/16	0.165
ACE inhibitor therapy, No. (%)	497 (20.5%)	361 (20.1%)	119 (20.8%)	17 (27.9%)	0.366
Angiotensin receptor blocker therapy, No. (%)	376 (15.5%)	275 (15.3%)	93 (16.2%)	8 (13.1%)	0.818
Calcium channel blocker, No. (%)	342 (14.1%)	252 (14.1%)	74 (12.9.6%)	14 (22.9%)	0.09
Beta blockers, No. (%)	387 (16.0%)	297 (16.6%)	77 (13.4%)	13 (21.3%)	0.104
Diuretic, No. (%)	439 (18.1%)	321 (17.9%)	108 (18.8%)	10 (16.4%)	0.828
Lipid-lowering drugs, No. (%)	280 (11.5%)	206 (11.5%)	67 (11.7%)	7 (11.5%)	0.992

Data are means ± SD. Triglyceride levels were log transformed for statistical analysis, but values in the Table represent back transformation to the original scale. Categorical variables were compared by χ^2^ test. Differences of continuous variables between groups were tested by a general linear model. P values refer to results after adjustment for sex and age

*ACE* angiotensin converting enzyme; *BMI* body mass index; *SBP* systolic blood pressure; *DBP* = diastolic blood pressure; *HDL* high density lipoprotein; *NGT* normal glucose tolerance

^*^P values refer to results after adjustment for sex

The clinical characteristics of the study group after stratification according to *TRIB3* Q84R genotype, are also presented in Table 1. The genotype distributions were in Hardy–Weinberg equilibrium (*P* > 0.05). No significant differences among the three genotypes were observed in relation to age, sex, BMI, waist circumference, smoking habit, eGFR, blood pressure, pulse pressure, heart rate, rate pressure product, and treatment with antihypertensive drugs. Similarly, we did not observe significant differences among the three genotypes in metabolic parameters including fasting glucose concentrations, total cholesterol, HDL cholesterol and triglycerides levels, and treatment with lipid-lowering drugs (Table 1). By contrast, fasting glucose levels (*P* = 0.02) and HOMA-IR index (*P* = 0.03) of insulin resistance were significantly associated with TRIB3 Q84R genotype (Table 1).

**Fig. 1:**
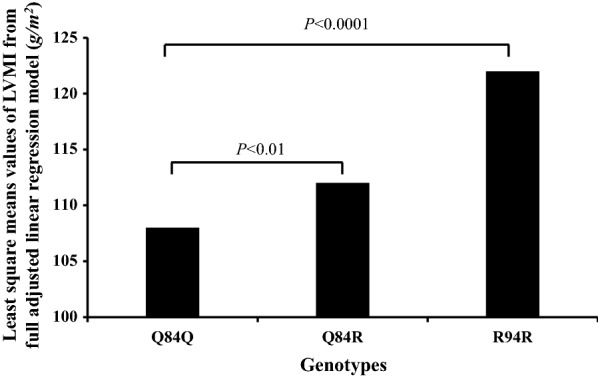
Least square means values of LVMI from adjusted linear regression model according to TRB3 genotype

Echocardiographic parameters of the study population are reported in Table 2. After adjusting for age and sex, both LV mass (LVM) and LV mass index (LVMI) were significantly increased in individuals carrying the QR (*P* = 0.03) and RR (*P* < 0.0001) genotype as compared with the QQ homozygous group (Table 2). The association between LVMI and the QR and RR genotype remained significant after adjusting for blood pressure, smoking habit, fasting glucose levels, glucose tolerance status, anti-hypertensive treatments, and lipid-lowering therapy (QR vs. QQ, *P* = 0.01; RR vs. QQ, *P* < 0.0001) (Fig. 1). Moreover, individuals carrying the RR genotype showed significantly higher values of left ventricular cavity size, expressed by left ventricular end-diastolic diameter (LVEDD), LV end-diastolic volume normalized by body surface area (LVEDVI), and posterior wall thickness (PWT) as compared to QQ homozygous (Table 2). No significant differences among the three genotypes were observed in early to late diastolic trans-mitral flow velocity (E/A) ratio, an index of diastolic function, and in LV ejection fraction (Table 2).

**Table 2:** Echocardiographic parameters of the study subjects stratified according to TRB3 genotypes

Variables	Whole study group	Q84Q	Q84R	R84R	*P*	*P* Q84R vs. Q84Q	*P* R84R vs. Q84Q
LV end-diastolic diameter (LVEDD) (cm)	4.94 ± 0.55	4.93 ± 0.54	4.96 ± 0.57	5.07 ± 0.61	0.09	0.39	0.04
LV end-diastolic volume (LVEDVI) (ml/m^2^)	66.9 ± 21.1	66.4 ± 20.0	68.1 ± 23.2	72.8 ± 28.3	0.05	0.24	0.02
Interventricular septal thickness (IVS) (cm)	1.08 ± 0.19	1.07 ± 0.19	1.09 ± 0.20	1.12 ± 0.18	0.173	0.284	0.104
Posterior wall thickness (PWT) (cm)	0.91 ± 0.16	0.90 ± 0.16	0.92 ± 0.18	0.96 ± 0.16	0.020	0.134	0.013
LV mass (g)	206 ± 66	203 ± 62	211 ± 74	232 ± 85	< 0.0001	0.032	< 0.0001
LV mass index (LVMI) (g/m^2^)	109 ± 32	108 ± 30	113 ± 35	125 ± 44	< 0.0001	0.033	< 0.0001
E/A ratio	0.98 ± 0.35	0.98 ± 0.34	0.98 ± 0.39	0.88 ± 0.20	0.198	0.458	0.117
Ejection fraction (%)	71 ± 8	71 ± 7	71 ± 8	70 ± 8	0.375	0.365	0.253

Data are means ± SD. Differences of continuous variables between groups were tested by a general linear model with post hoc Fisher's least significant difference correction for pairwise comparisons. *P* values refer to results after adjustment for age, and sex. *LV* left ventricular

To estimate the independent contribution of the *TRIB3* Q84R genotype to LVMI, we carried out a linear regression analysis in a model including potential modulators of LVMI such as sex, age, BMI, systolic and diastolic blood pressure, smoking habit, fasting plasma glucose, glucose tolerance status, antihypertensive treatments, and lipid-lowering therapy. Comparison of standardized coefficients allowed the determination of the relative strength of the association of each trait with LVMI (listed from strongest to weakest): male sex (β = 0.300 ± 1.215 *P* < 0.0001), age (β = 0.283 ± 0.046, *P* < 0.0001), systolic blood pressure (β = 0.193 ± 0.041, *P* < 0.0001), antihypertensive treatments (β = 0.135 ± 0.048, *P* < 0.0001), *TRIB3* Q84R polymorphism (β = 0.070 ± 0.048, *P* < 0.0001), and BMI (β = 0.055 ± 0.093, *P* = 0.002). The model accounted for 30.1% of the variation in LVMI. Smoking habit, diastolic blood pressure, fasting plasma glucose, glucose tolerance status, and lipid-lowering therapy were not independently associated with LVMI.

## Discussion

Prior studies in animal models have shown that increased cardiac expression of TRIB3 has a pathogenic role in inducing LVM [28, 29]. Furthermore, a gain-of-function variant in *TRB3* gene that has been identified and extensively characterized (i.e. Q84R, where arginine replaces glutamine at position 84; rs2295490) has been associated with in vitro [30, 31] and in vivo vascular insulin resistance [31] and earlier onset of myocardial infarction [27, 41]. These observations have provided the rationale for addressing the question of whether the *TRIB3* Q84R polymorphism could be associated with LVMI. We found that, in a group of 2426 adult White individuals, LVMI progressively and significantly increased from 108 to 113, to 125 g/m^2^ in Q84Q, Q84R, and R84R individuals, respectively, even after adjustment for several confounding cardio-metabolic risk factors such as sex, age, smoking habit, blood pressure, fasting plasma glucose, glucose tolerance status, antihypertensive treatments, and lipid-lowering therapy.

As to the mechanisms by which the *TRIB3* R84 variant induces the observed increase in left ventricular mass, we like to hypothesize that it is a consequence of increased TRIB3 function in cardiomyocytes causing a selective insulin resistance resulting in impaired activation of cardioprotective PI3K/Akt-dependent insulin signaling pathway, due to enhanced binding to Akt by the gain-of-function *TRIB3* R84 variant, and enhanced activation of the growth-promoting ras/mitogen-activated protein kinase (MAPK)-dependent pathway, which has been associated with cardiac hypertrophy [42]. Indeed, this hypothesis is supported by the results of prior studies carried out in primary HUVECs naturally carrying the TRIB3 Q84 or R84 variant showing a constitutive MAPK kinase-MAPK activation in parallel with markedly reduced insulin-induced Akt activation in endothelial cells carrying the TRIB3 Q84R or R84R genotype compared with those carrying the Q84Q genotype [31]. Additionally, in a rat model of type 2 diabetes, *TRIB3* gene silencing reverted myocardial remodeling by restoring Akt activation and reducing the increased activation of MAPK [28]. Thus, it is tempting to speculate that the *TRIB3* R84 variant, because of its gain-of-function, behaves on MAPK stimulation similar to TRIB3 overexpression and is, thus, a positive modulator of this signal transduction pathway.

The current study has some strengths including the strong biological plausibility based on the results of previous in vitro studies using primary HUVECs naturally carrying the TRIB3 Q84 or R84 variant, the relatively large sample size comprising both men and women, the exclusion of confounding clinical conditions potentially affecting LV mass, the detailed phenotype characterization of participants by trained physicians in a clinical setting that allowed to directly assess the cardio-metabolic characteristic of individuals (no self-reported data were used), the homogeneity of study subjects recruited among Italians born in Southern Italy, a population that shows limited substructure in a principal component analysis of Genome Wide Association Studies data [43], and the echocardiographic assessments performed by a single skilled examiner, who was blinded to the clinical and laboratory results of participants.

Nonetheless, the present study must be interpreted within the context of its possible limitations. First, the study lacks of replication using an independent sample, and, therefore, the results should be considered explorative in nature although the hypothesis tested is biologically plausible and the present results are consistent with the data obtained in animal models. Secondly, the cross-sectional design of the study allows to show only an association with prevalent, but not incident LV mass. Thus, although we were able to observe an highly significant effect of the *TRIB3* Q84R polymorphism on LVMI, our results need replication in prospective studies before it can be considered as validated. Additionally, the study subjects were outpatients recruited at a referral university hospital, representing individuals at enhanced risk for cardio-metabolic disease, and, therefore, the results of this study may not be extendible to the general population. Moreover, the study encompassed only non-diabetic individuals, thus excluding from the analysis patients with type 2 diabetes who are at very high risk of cardiovascular disease. Furthermore, the results of previous studies assessing the functional impact of the *TRIB3* Q84R variant were obtained in venous endothelial cells, a model which does not necessarily resemble that of human cardiomyocytes. However, the possibility that the *TRIB3* Q84R variant does not affect human cardiomyocytes is unlikely on light of the findings that studies in primary human endothelial cells [30, 31] and in isolated human pancreatic islets naturally carrying the *TRIB3* Q84R variant, as well as in human hepatoma cells transfected with and expressing the *TRIB3* Q84R variant [41] have very consistently reported that *TRIB3* R84 acts as a gain-of-function variant that alters insulin signaling, and, consequently, cellular specific insulin actions. Finally, the present findings may apply only to White individuals of European ancestry, and should not be extended to other ethnic groups since there are differences in cardio-metabolic risk among different ethnic groups likely due to socio-demographic, lifestyle, anthropometric, and genetic characteristics. Thus, our findings are hypothesis generating that require confirmation by further studies including individuals of other ethnic groups. Nevertheless, we consider our findings important in attempting to understand the pathophysiological interaction between the *TRIB3* Q84R polymorphism and cardiovascular disease.

## Conclusions

We supply evidences that the gain-of-function *TRIB3* Q84R variant is significantly associated with left ventricular mass in a large sample of White nondiabetic individuals of European ancestry. These original findings might help elucidate the molecular mechanism linking insulin resistance/hyperinsulinemia to altered myocardial structure.

## Data Availability

All data generated or analysed during this study are included in this published article.

## Abbreviations

ADA: American Diabetes Association
BMI: Body mass index
BSA: Body surface area
ESRD: End-stage renal disease
LVESV: End-systolic volume
HBsAg: Hepatitis B surface antigen
HCV: Hepatitis C virus
HUVECs: Human umbilical vein endothelial cells
IVS: Interventricular septum thickness
LVEDD: Left ventricular end-diastolic diameter
LVEDV: LV end-diastolic
LVEDVI: LV end-diastolic volume index
LVM: Left ventricular mass
LVMI: LVM index
MT: Metallothionein
NGT: Normal glucose tolerance
PHLPP: PH domain leucine-rich repeat protein phosphatase
PTEN: Phosphatase and tensin homologue
PI3K: Phosphatidylinositol3-kinase
PWT: Posterior wall thickness
Akt: Protein kinase B
PTP1B: Protein tyrosine phosphatase 1B
MAPK: Ras/mitogen-activated protein kinase
TRIB3: Tribbles homologue 3
